# HbA1c and brain health across the entire glycaemic spectrum

**DOI:** 10.1111/dom.14321

**Published:** 2021-02-14

**Authors:** Victoria Garfield, Aliki‐Eleni Farmaki, Sophie V. Eastwood, Rohini Mathur, Christopher T. Rentsch, Krishnan Bhaskaran, Liam Smeeth, Nish Chaturvedi

**Affiliations:** ^1^ MRC Unit for Lifelong Health and Ageing at UCL Institute of Cardiovascular Science, University College London London UK; ^2^ Department of Non‐communicable Disease Epidemiology London School of Hygiene & Tropical Medicine London UK

**Keywords:** cohort study, diabetes complications, observational study, population study, type 2 diabetes

## Abstract

**Aim:**

To understand the relationship between HbA1c and brain health across the entire glycaemic spectrum.

**Materials and Methods:**

We used data from the UK Biobank cohort consisting of 500,000 individuals aged 40–69 years. HbA1c and diabetes diagnosis were used to define baseline glycaemic categories. Our outcomes included incident all‐cause dementia, vascular dementia (VD), Alzheimer's dementia (AD), hippocampal volume (HV), white matter hyperintensity (WMH) volume, cognitive function and decline. The reference group was normoglycaemic individuals (HbA1c ≥35 & <42 mmol/mol). Our maximum analytical sample contained 449,973 individuals with complete data.

**Results:**

Prediabetes and known diabetes increased incident VD (HR 1.54; 95% CI = 1.04, 2.28 and HR 2.97; 95% CI = 2.26, 3.90, respectively). Known diabetes increased all‐cause and AD risk (HR 1.91; 95% CI = 1.66, 2.21 and HR 1.84; 95% CI = 1.44, 2.36, respectively). Prediabetes and known diabetes elevated the risks of cognitive decline (OR 1.42; 1.48, 2.96 and OR 1.39; 1.04, 1.75, respectively). Prediabetes, undiagnosed and known diabetes conferred higher WMH volumes (3%, 22% and 7%, respectively) and lower HV (36, 80 and 82 mm^3^, respectively), whereas low‐normal HbA1c had 1% lower WMH volume and 12 mm^3^ greater HV.

**Conclusion:**

Both prediabetes and known diabetes are harmful in terms of VD, cognitive decline and AD risks, as well as lower HV. Associations appeared to be somewhat driven by antihypertensive medication, which implies that certain cardiovascular drugs may ameliorate some of the excess risk. Low‐normal HbA1c levels, however, are associated with more favourable brain health outcomes and warrant more in‐depth investigation.

## 1 INTRODUCTION

Type 2 diabetes and, more generally, hyperglycaemic states, have been associated with poorer cognitive function (such as learning and memory),^1^, ^2^ increased risk of dementia^2^, ^3^ and alterations in key brain structures, particularly the hippocampus.^4^ However, it is also important to explore how low‐normal levels (vs. normal glycaemic levels) of HbA1c relate to brain health outcomes, a subject that has not been investigated in a population‐based study to date. A previous paper explored the cross‐sectional association between baseline diabetes and two cognition measures in the UK Biobank (UKB) (reaction time [RT] and visual memory).^5^ The authors found that diabetes was associated with poorer scores on the RT test, but paradoxically, better scores on the visual memory test. They did not explore other brain health outcomes or lesser glycaemic states.

Memory loss is the most conclusively reported adverse effect of hyperglycaemia on cognitive function,^6^ yet hyperglycaemia is also associated with poorer processing speed, attention, concentration and executive functions.^7^ Hippocampal atrophy is a crucial feature of age‐related memory loss and the hippocampus is reportedly more vulnerable to the neurotoxic consequences of diabetes.^8^, ^9^ Evidence relating diabetes to the presence and progression of white matter hyperintensities (WMHs) is equivocal,^10^ but some research suggests that those with diabetes have greater volumes of WMH.^11^, ^12^ Although there have been numerous studies in this area, the role of glycaemia in brain health across the entire glycaemic spectrum remains unclear. In particular, no studies have investigated how lesser hyperglycaemic states relate to these outcomes, as most studies have focused on diagnosed diabetes.

Thus, our aim was to investigate, in a single large‐scale study, the associations between five glycaemic states across the entire spectrum (low‐normal HbA1c, normoglycaemia, prediabetes, undiagnosed diabetes and known diabetes) and a number of brain health outcomes including Alzheimer's dementia (AD) risk, vascular dementia (VD) risk, baseline cognitive function and cognitive decline, hippocampal volume and WMH volume in the UKB. We hypothesized that those individuals with increasingly higher HbA1c levels would have poorer outcomes compared with those individuals with normal glycaemic levels.

## 2 METHODS

### 2.1 Sample

**FIGURE 1:**
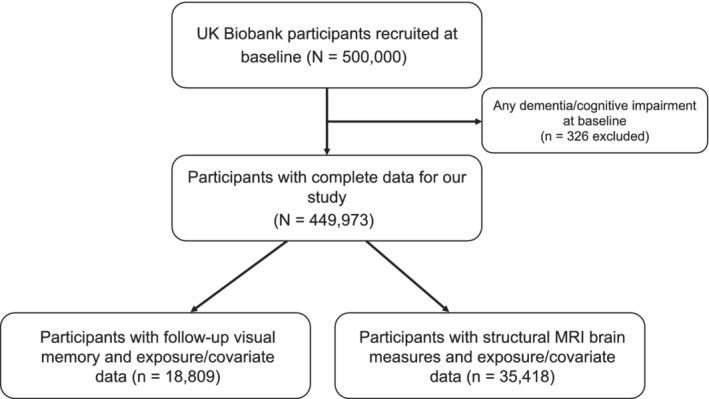
Study design

Full details of the UKB cohort have been described elsewhere.^13^ Briefly, the UKB consists of data from approximately 500,000 men and women from the general UK population aged 40–69 years at baseline during 2006–2010 (Supplementary Material S1; see the supporting information). Figure 1 depicts our study design.

### 2.2 Informed consent and ethical approval

The UKB received ethical approval from the North West Multicentre Research Ethics Committee and informed consent was obtained from participants.

### 2.3 Type 2 diabetes

Exposure status was defined using baseline data on type 2 diabetes (diabetes) and HbA1c (Supplementary Material S1). Diabetes was defined using an algorithm of self‐report, doctor diagnosis and/or medication; this algorithm has been validated against primary care data.^14^ In this study, values greater than 200 mmol/mol were excluded (n = 5) as they were considered to be outliers and clinically implausible. For our analyses we divided participants into the following categories: known diabetes, undiagnosed diabetes (≥48 mmol/mol), prediabetes (42 ≤ 48 mmol/mol), normoglycaemic (≥35 and <42 mmol/mol) and low HbA1c (<35 mmol/mol), based on criteria by Ginde et al.^15^

### 2.4 Cognitive function

We pragmatically selected two measures with adequate sample sizes to represent distinct cognitive domains, namely, RT and visual memory. In the visual memory test, respondents had to identify matches from six pairs of cards after memorizing their positions on the screen. The number of incorrect matches (errors made) was then recorded, whereby a higher number indicated poorer visual memory. Participants also completed a timed assessment of symbol matching, which was similar to the card game ‘Snap’. RT was measured as the mean time (in ms) taken to correctly identify matches from trials that had matching symbol pairs. A higher score (longer time) indicates slower RTs. As per Lyall et al.,^16^ RT was transformed using a log transformation (ln) and visual memory was transformed using a ln + 1 equation (because of zero‐value inflation). The total sample size for the RT and visual memory baseline analyses was 449,973.

### 2.5 Neuroimaging outcomes

Structural brain MRI scans have been performed in a subsample of UKB participants using standard protocols (Supplementary Material S1).^17^ Postprocessed measures (provided by the UKB) used in this study included hippocampal volume (mm^3^, normalized for head size) and total volume of WMH (mm^3^). WMH volume was log‐transformed as it was positively skewed. Thus, we report exponentiated betas for this outcome to ease interpretation. The maximum sample size for these outcomes in our study was 35,418.

### 2.6 Dementia

Dementia at baseline was captured using International Classification of Diseases (ICD)‐10 codes in linked hospital episode statistics (HES) data. Incident dementia was algorithmically defined with the method described in Wilkinson et al.,^18^ which was based on linked UK hospital admission, mortality and primary care data. Coded diagnoses were compared against clinical expert adjudication of full‐text medical records. Here, we focus on all‐cause dementia (n = 2023) (see Supplementary Material S1), VD (n = 412) and AD (n = 749). Frontotemporal dementia cases were only included in all‐cause dementia analyses (n = 95).

### 2.7 Cognitive decline

Using data from a subset of participants who had both baseline and follow‐up measures of cognitive function, cognitive decline was determined using the standardized regression‐based method.^19^ This included regressing follow‐up visual memory on baseline visual memory, as well as age, sex, years of education and time between the two assessments. Those whose standardized residual was greater than (absolute value) 1.96 (0.05 type 1 error rate) were designated as having cognitive decline. Only a proportion of the UKB participants had follow‐up visual memory data and complete covariate data (n = 18 809). This was because a subsample underwent repeat cognitive assessment between the summers of 2012 and 2013, all of whom lived within 35 km of the Stockport (UK) UKB centre. The response rate was 21% to the email or letter invitation.

### 2.8 Covariates

Demographics such as age (years), sex, ethnicity (White European, Asian/Asian British, Black/Black British, Other), deprivation (quintiles of Townsend deprivation index, from ‘least deprived’ to ‘most deprived’) and educational attainment (derived as years of full‐time education completed, as per qualifications based on coding from the International Standard Classification of Education^20^) were included. Health behaviours included smoking status (never, current smoker and ex‐smoker). Health measures included body mass index (BMI) in kg/m^2^, baseline cardiovascular disease (CVD; assigned using baseline self‐report, nurse interview and linked hospital inpatient data from 2006 to 2010), antihypertensive medication and statin use. Medications were captured and classified according to British National Formulary chapters.

### 2.9 Exclusion criteria

We excluded those who had dementia or cognitive impairment prior to their recorded date of baseline assessment (2006–2010), as captured by self‐report, nurse interview or HES.

### 2.10 Missing data

There were missing data across several variables, all of which had less than 10% missingness, and for this reason we used complete case analysis for this study. The missing data were as follows: ethnicity (n = 2275), BMI (n = 3260), RT (n = 5776), visual memory (n = 4627), deprivation (n = 623), smoking (n = 1918), HbA1c (n = 34 594), antihypertensives and statins (n = 8589) and educational attainment (n = 9133).

### 2.11 Statistical analyses

Analyses were performed in RStudio version 1.1.456 and STATA version 15.

### 2.12 Modelling approach

#### 2.12.1 Cross‐sectional analyses

##### Cognitive function and neuroimaging outcomes

In the cross‐sectional analyses, glycaemia was entered as an exposure and four linear regressions were fitted to explore the relationship with baseline cognition outcomes (RT and visual memory). Model 1 consisted of adjustment for demographic measures (age + sex + deprivation + educational attainment + ethnicity), while Model 2 was additionally adjusted for standard cardiovascular risk factors (smoking + BMI + CVD + antihypertensives + statins). Our modelling approach was identical for neuroimaging outcomes (hippocampal volume and volume of WMH).

#### 2.12.2 Longitudinal analyses

##### Dementia

Cox proportional hazards models were used to examine the relationships between glycaemia and all‐cause dementia, AD and VD. The time scale was time since study entry and participants were followed up until 31 March 2017. The same modelling strategy was used, as described above. The proportional hazards assumption was assessed using the global test to evaluate the interaction of each covariate with time, alongside Schoenfeld residuals.

##### Cognitive decline

Only 4% of UKB participants underwent follow‐up cognition testing, so our analyses of cognitive decline were restricted to this subpopulation. Logistic regression was used to investigate the association between glycaemia and binary cognitive decline, with the same modelling strategy as above.

## 3 RESULTS

### 3.1 Sample characteristics

A total of 449,973 individuals were included in the study, of whom 210,309 had low‐normal HbA1c levels, 198,969 had normoglycaemic levels, 15,229 had prediabetes, 3279 had undiagnosed diabetes and 22,187 had known diabetes. Those with prediabetes and known diabetes were older than the other groups. Those with diabetes (undiagnosed and known) were more probable to be ex‐smokers, reside in the most deprived quintile and have higher BMI values (Table 1). Those with known diabetes were most probable to be taking antihypertensives and statins at baseline and had the highest prevalence of CVD.

**TABLE 1:** Baseline characteristics and outcomes across the glycaemic spectrum, N = 449,973

	Low HbA1c (210,309)	Normoglycaemic (198,969)	Prediabetes (15,229)	Undiagnosed (3279)	Known (22,187)	*p‐value*
Age, years: mean (SD)	54.4 (8.2)	58.1 (7.5)	60.1 (6.8)	58.5 (7.3)	59.8 (7.1)	<.001
Men, N (%)	94 447 (45)	87 597 (44)	7205 (47)	1967 (60)	13 862 (62)	
Education, years: mean (SD)	15.5 (4.9)	14.7 (5.2)	13.8 (5.3)	13.9 (5.2)	13.7 (5.3)	<.001
Ethnicity, N (%)						<.001
White European	204 186 (97.1)	189 305 (95.1)	13 435 (88.2)	2837 (86.5)	19 880 (89.6)	
South Asian	1514 (0.7)	2904 (1.5)	519 (3.4)	165 (5)	1003 (4.5)	
African Caribbean	1499 (0.7)	2636 (1.3)	670 (4.4)	148 (4.5)	558 (2.5)	
Mixed or other	3110 (1.5)	4124 (2.1)	605 (4)	129 (3.9)	746 (3.4)	
Deprivation, N (%)						<.001
Least deprived	44 865 (21)	40 688 (20)	2583 (17)	501 (15)	3354 (15)	
Second least deprived	43 902 (21)	40 490 (20)	2769 (18)	531 (16)	3728 (17)	
Median deprivation level	42 853 (20)	40 498 (20)	2824 (18)	606 (18)	4105 (18)	
Second most deprived	41 925 (20)	39 377 (20)	3198 (21)	676 (21)	4672 (21)	
Most deprived	36 764 (17)	37 916 (19)	3855 (25)	965 (29)	6328 (28)	
Smoking, N (%)						<.001
Never smoker	148 515 (71)	127 610 (64)	8539 (56)	1824 (56)	12 188 (55)	
Current smoker	16 908 (8)	24 476 (12)	2474 (16)	500 (15)	2386 (11)	
Ex‐smoker	44 886 (21)	46 883 (24)	4216 (28)	955 (29)	7613 (34)	<.001
BMI, kg/m^2^: mean (SD)	26.5 (4.2)	27.6 (4.7)	30.3 (5.5)	32 (5.7)	31.4 (5.8)	<.001
HbA1c, mmol/mol: mean (SD)	32.1 (2.3)	37.4 (1.8)	43.8 (1.5)	58.7 (15.1)	53.1 (13.9)	<.001
HbA1c, %: mean (SD)	5.1 (0.2)	5.6 (0.2)	6.2 (0.1)	7.5 (1.4)	7 (1.3)	<.001
Statins N (%)	18 450 (9)	36 447 (18)	5195 (34)	983 (30)	17 022 (77)	<.001
Antihypertensives, N (%)	28 757 (14)	43 287 (22)	5731 (38)	1127 (34)	14 435 (65)	<.001
Baseline CVD, N (%)	7974 (4)	14 559 (7)	2364 (15)	450 (14)	4803 (22)	<.001
Cognitive function at baseline						
RT, ms: mean (SD)	545.7 (108.9)	565.4 (116)	584.1 (129.3)	579.5 (127)	587.5 (129.6)	<.001
VM, incorrect matches: mean (SD)	4.0 (3.2)	4.3 (3.4)	4.4 (3.6)	4.4 (3.5)	4.3 (3.6)	<.001
Incident dementia						
All‐cause dementia, N (%)	678 (0.3)	920 (0.5)	110 (0.7)	16 (0.5)	299 (1.3)	<.001
AD, N (%)	267 (0.1)	349 (0.2)	32 (0.2)	5 (0.2)	96 (0.4)	<.001
VD, N (%)	110 (0.1)	165 (0.1)	30 (0.2)	5 (0.2)	102 (0.5)	<.001
Follow‐up subsample of n = 18,809						
Cognitive decline, N (%)	375 (4)	361 (4)	32 (6)	8 (0.2)	42 (6)	<.001
Imaging subsample of n = 35,418						
n	9978	7669	400	79	452	
WMHV, mm^3^: median (IQR)	2268 (3187)	2965 (4449)	3948 (5216)	4275 (7183)	4089 (6252)	<.001
HV, mm^3^: mean (SD)	3884.3 (432.3)	3817.3 (432.1)	3766.3 (453.3)	3864.3 (570.9)	3766.1 (445.9)	<.001

Abbreviations: AD, Alzheimer's dementia; BMI, body mass index (kg/m^2^); CVD, cardiovascular disease; HV, hippocampal volume; IQR, interquartile range; RT, reaction time; SD, standard deviation; VD, vascular dementia; VM, visual memory; WMHV, white matter hyperintensity volume.

*Note*: Low HbA1c < 35 mmol/mol; normoglycaemic 35 ≤ 42 mmol/mol; prediabetes 42 ≤ 48 mmol/mol; undiagnosed diabetes ≥ 48 mmol/mol.

### 3.2 Cross‐sectional results

#### 3.2.1 Cognitive function and neuroimaging outcomes

Those with low‐normal HbA1c levels had RTs that were no different to those of the normoglycaemic group. However, both undiagnosed and known diabetes were associated with 2% slower RTs, while, upon multivariate adjustment, prediabetes was associated with 1% slower RTs (Table 2). Low‐normal HbA1c and undiagnosed diabetes were not associated with visual memory scores, but those with known diabetes made 3% fewer errors compared with the normoglycaemic group (Table 2).

**TABLE 2:** Association between glycaemia and baseline cognitive function, N = 449,973

	Reaction time	Visual memory
Group	Expß (95% CI)	Expß (95% CI)
Model 1		
Low HbA1c	1.00 (0.99, 1.00)	1.00 (1.00, 1.00)
Prediabetes	1.01 (1.01, 1.01)	0.99 (0.98, 1.00)
Undiagnosed T2D	1.01 (1.01, 1.02)	0.99 (0.96, 1.01)
Known T2D	1.02 (1.01, 1.02)	0.97 (0.96, 0.98)
Model 2		
Low HbA1c	1.00 (0.99, 1.00)	1.00 (0.99, 1.00)
Prediabetes	1.01 (1.01, 1.01)	1.00 (0.98, 1.01)
Undiagnosed T2D	1.02 (1.01, 1.02)	1.00 (0.98, 1.03)
Known T2D	1.02 (1.01, 1.02)	0.97 (0.96, 0.98)

Abbreviations: 95% CI, 95% confidence interval; BMI, body mass index; CVD, cardiovascular disease; Exp(ß), exponentiated beta; T2D, type 2 diabetes. Model 1 = adjusted for age + sex + deprivation + ethnicity + educational attainment. Model 2 = Model 1 + BMI + CVD + statins + antihypertensives + smoking.

**FIGURE 2:**
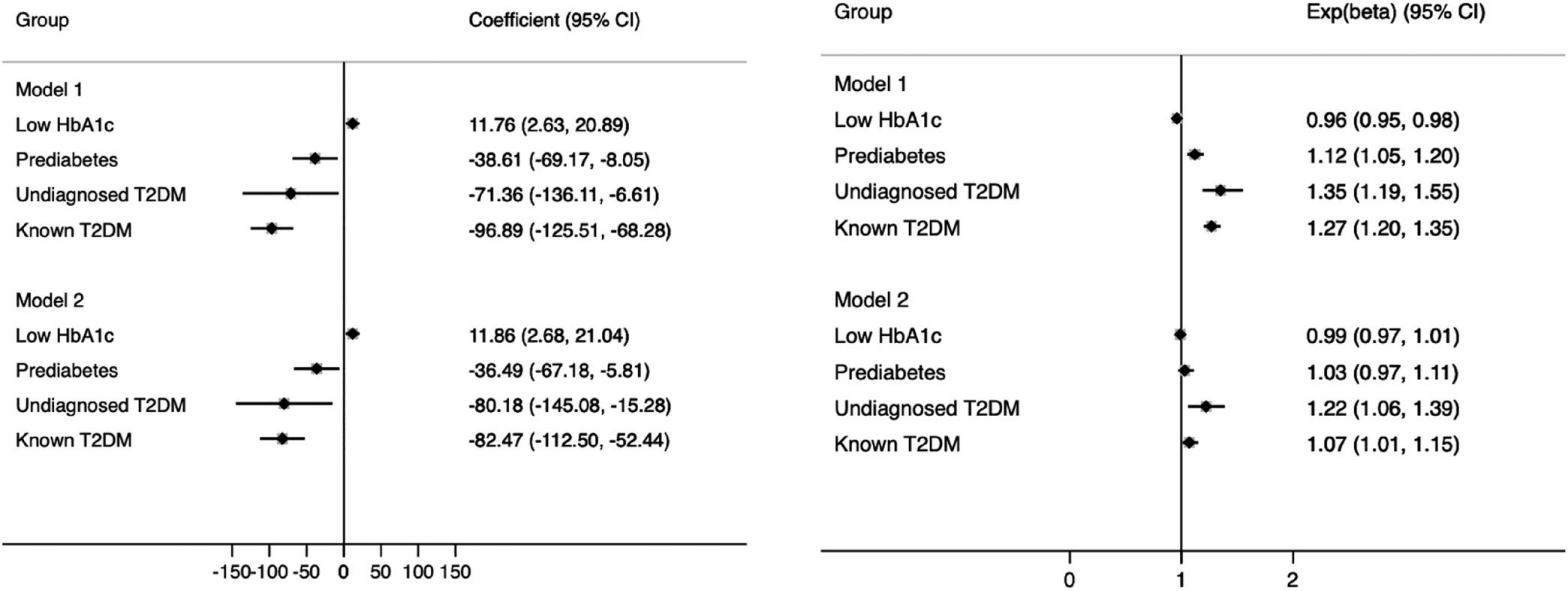
Association between glycaemia and cognitive decline in a UK Biobank subsample (N = 18,809). Model 1 = adjusted for age + sex + deprivation + ethnicity + educational attainment. Model 2 = Model 1 + BMI + CVD + statins + antihypertensives + smoking. 95% CI, 95% confidence interval; BMI, body mass index; CVD, cardiovascular disease

Low‐normal HbA1c was associated with lower WMH volume and greater hippocampal volume compared with normoglycaemic individuals. Prediabetes, undiagnosed and known diabetes were associated with higher WMH volume and lower hippocampal volume (Figure 2). Multivariable adjustment, specifically the addition of antihypertensive therapy, markedly attenuated associations with WMH volume for prediabetes and known diabetes, but less so for undiagnosed diabetes. Thus prediabetes, undiagnosed diabetes and known diabetes were associated with greater WMH volumes (3%, 22% and 7%, respectively) and smaller hippocampal volumes (36 mm^3^, 80 mm^3^, 82 mm^3^) in fully adjusted models. Those with low‐normal HbA1c had 1% lower WMH volume (which did not reach conventional levels of statistical significance upon multiple adjustment) and 12 mm^3^ larger hippocampal volumes than normoglycaemic individuals.

### 3.3 Longitudinal results

#### 3.3.1 Dementia

**FIGURE 3:**
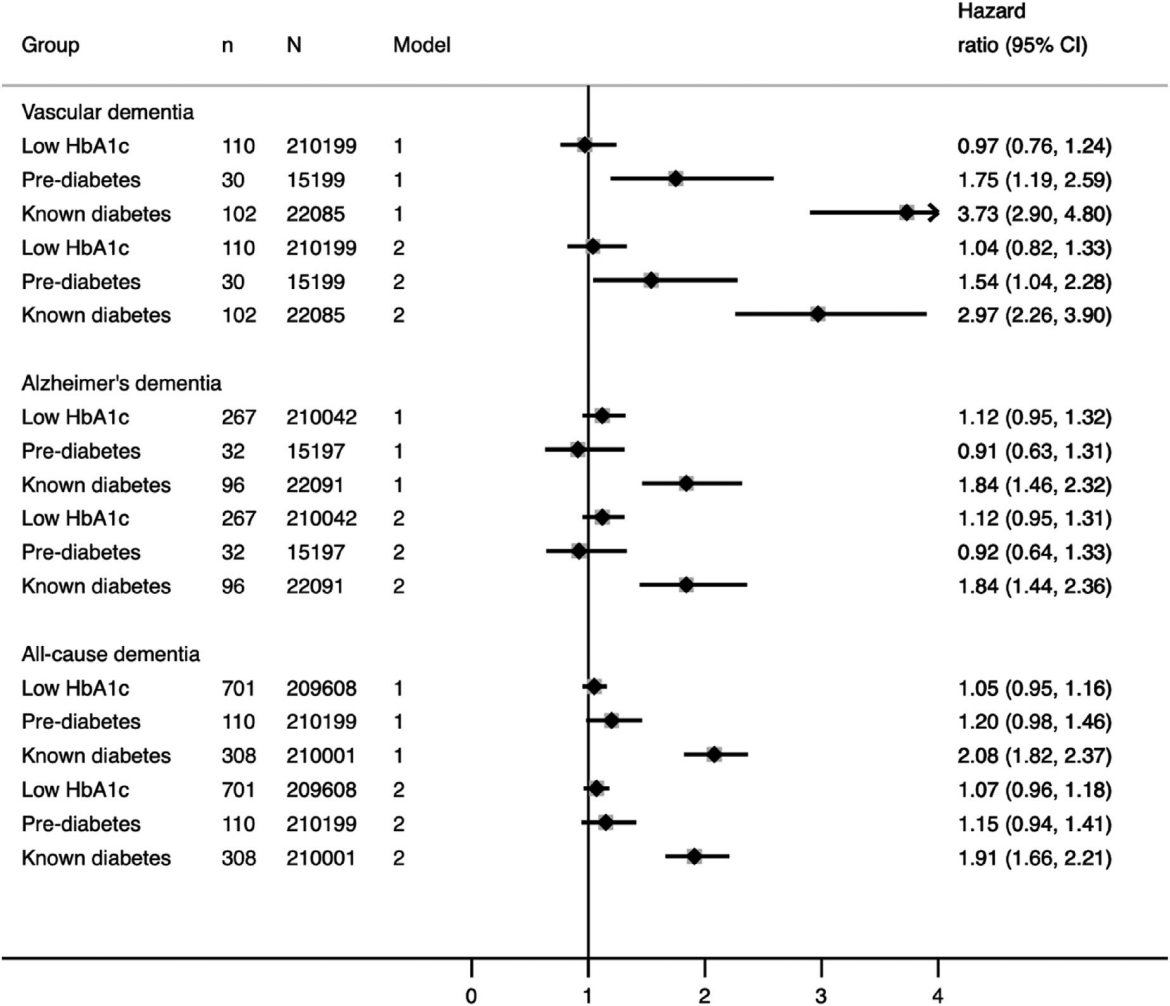
Association between glycaemia and incident all‐cause, Alzheimer's and vascular dementia in the UK Biobank (N = 449,973). Model 1 = adjusted for age + sex + deprivation + ethnicity + educational attainment. Model 2 = Model 1 + BMI + CVD + statins + antihypertensives + smoking. 95% CI, 95% confidence interval; BMI, body mass index; CVD, cardiovascular disease; T2D, type 2 diabetes

We do not present results from the undiagnosed diabetes group, as the number of cases for all‐cause dementia, AD and VD was less than 20. Prediabetes and low‐normal HbA1c were not associated with all‐cause dementia or AD in basic or fully adjusted models (Figure 3). However, known diabetes was strongly associated with excess all‐cause dementia and AD risk upon minimal adjustment and this remained robust in fully adjusted models (HR 1.91, 95% CI = 1.66, 2.21 and HR 1.84, 95% CI = 1.44, 2.36, respectively). People with prediabetes had elevated risks of VD, as did those with known diabetes (HR 1.75, 95% CI = 1.19, 2.59 and HR 3.73, 95% CI = 2.90, 4.80, respectively; Figure 3), but low‐normal HbA1c was not associated with VD (Figure 3).

Adjustment for health‐related measures attenuated the associations between glycaemia and VD. However, this remained large at a 54% increased risk of VD for prediabetes and an almost threefold excess risk for known diabetes. In multivariate models, the key factor responsible for accounting for excess risk for both prediabetes and known diabetes was antihypertensive therapy. Model 1 HRs were 1.75 (95% CI = 1.19, 2.59) for prediabetes and 3.73 (95% CI = 2.90, 4.80) for known diabetes. Additional adjustment for antihypertensive therapy only (in addition to Model 1) resulted in HRs of 1.61 (95% CI = 1.09, 2.39) for prediabetes and 3.04 (95% CI = 2.34, 3.95) for known diabetes. We also performed sensitivity analyses for all‐cause dementia, AD and VD, in which we included both systolic blood pressure alongside antihypertensives in fully adjusted models. As the results remained qualitatively identical, albeit with less precision because of a smaller number of cases, we do not present these estimates. Additional analyses of confounding by age are provided in Table S1.

#### 3.3.2 Cognitive decline

**FIGURE 4:**
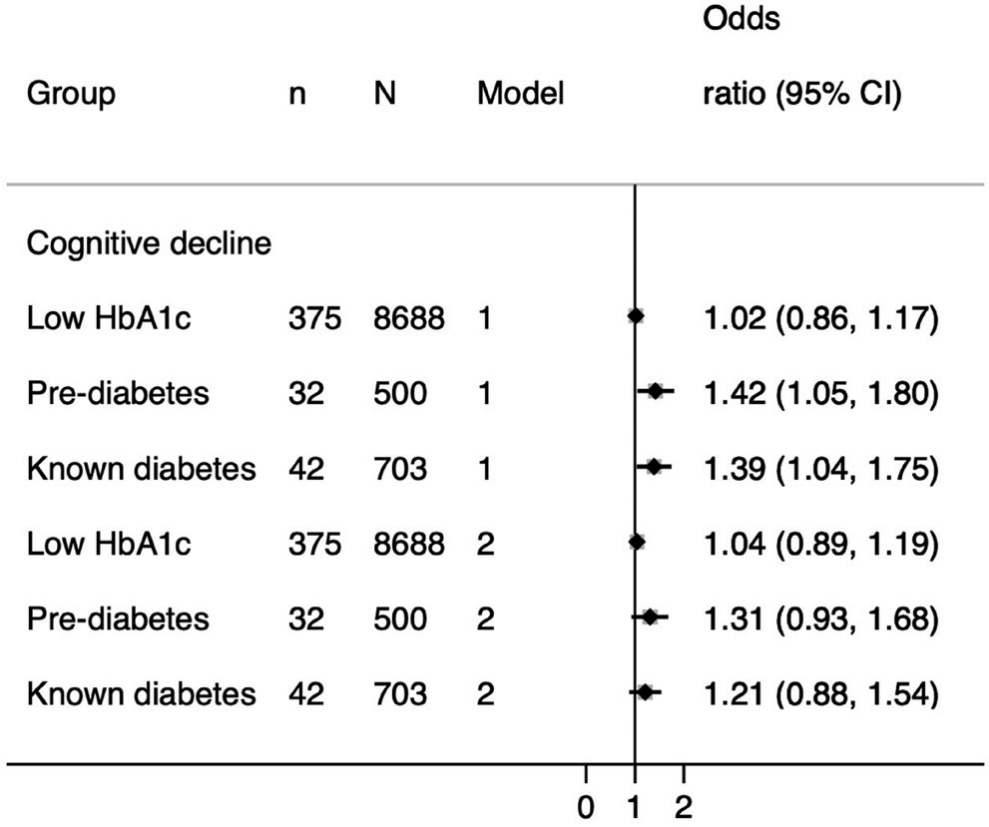
Association between glycaemia and cognitive decline in a UK Biobank subsample (N = 18,809). Model 1 = adjusted for age + sex + deprivation + ethnicity + educational attainment. Model 2 = Model 1 + BMI + CVD + statins + antihypertensives + smoking. 95% CI, 95% confidence interval; BMI, body mass index; CVD, cardiovascular disease

In Model 1 (demographics), prediabetes and known diabetes were associated with a somewhat greater risk of cognitive decline (Figure 4), but the 95% confidence intervals (CIs) around the odds ratios (ORs) were wide. However, in the fully adjusted model these associations became more pronounced, and prediabetes and known diabetes were associated with a 42% and 39% increased risk of cognitive decline, respectively. Upon close inspection of the model, we observed a strong relationship between BMI and cognitive decline, which suggested that those individuals with higher BMI values were less probable to experience cognitive decline (OR 0.97; 95% CI = 0.95, 0.99). This remained identical upon multivariate adjustment.

## 4 DISCUSSION

In this large sample of middle‐aged adults (age range 40‐69 years), we report four key findings. First, people with prediabetes and known diabetes have excess risks of clinically important outcomes (cognitive decline and dementia). Second, a key determinant of the excess risk of VD in association with hyperglycaemia is antihypertensive medication. Third, associations between hyperglycaemia and dementia are stronger for vascular dementia than for all‐cause dementia and AD. Fourth, we observed that low‐normal levels of glycaemia may be somewhat beneficial in relation to subclinical measures of brain health, such as certain neuroimaging outcomes.

We observed that prediabetes is associated with 1% slower RTs, whereas undiagnosed and known diabetes are associated with 2% slower RTs. This finding is supported by an early study of patients with diabetes who performed slower on an RT task compared with age‐matched controls.^21^ We show that there are apparent associations, at least cross‐sectionally, with prediabetes and undiagnosed diabetes compared with normoglycaemia. The association we observed between glycaemia and visual memory was somewhat paradoxical, as known diabetes was associated with 3% fewer incorrect matches on this task. It is possible, however, that other factors common to individuals with diabetes (e.g. the effects of medication on control of glycaemia) could perhaps confer some protection against poorer visual memory.

Another novel finding is that in minimally adjusted models low‐normal HbA1c levels were associated with greater hippocampal volume and lower WMH volume compared with normoglycaemic individuals. Participants with low‐normal HbA1c tended to be younger and healthier than those in the other groups, were less probable to be smokers, less probable to reside in higher quintiles of deprivation, had a lower prevalence of baseline CVD, and fewer were on statins or antihypertensives. Adjustment for these factors somewhat attenuated the relationship between low‐normal HbA1c and WMH volumes (from 4% to 1% and did not reach conventional levels of statistical significance), but this was not the case for hippocampal volume. This may, once again, suggest that distinct mediators operate in the association between glycaemia and AD, and atrophy of the brain, compared with factors that mediate the relationship between glycaemia and vascular brain damage. Although our findings preclude us from drawing any temporal or causal claims about this association, it is possible that in middle‐aged adults (aged approximately 54 years) without diabetes, HbA1c levels below 35 mmol/mol could confer some protection against hippocampal atrophy, as well as the presence of WMHs. However, these findings warrant replication to determine whether this is true and, if so, what the underlying mechanisms may be. Our results also indicate that pathways to brain health in association with persistently lower HbA1c in people without diabetes are probably different to those with bouts of hypoglycaemia in people with diabetes.

It is striking that in comparison with normoglycaemic individuals, prediabetes and known diabetes both increase the risk of VD, cognitive decline and, to a slightly lesser extent, all‐cause dementia and AD. A recent meta‐analysis suggests excess dementia risk in prediabetes^2^ but most studies do not make a direct comparison with people with established diabetes and have been restricted by small numbers of events. Risks of cognitive decline have been more extensively studied, with the majority identifying prediabetes as a high‐risk state, although few suggest that risks are close to established diabetes.^22^, ^23^ This has important implications for intervention. With greater numbers of individuals surviving to older age, avoidance, or at least postponement of dementia, is an increasing therapeutic concern. Therefore, much like the finding of excess CVD risks in people with prediabetes,^24^, ^25^ this result prompts consideration of identification and early intervention in such individuals.

Midlife hypertension increases dementia risk^26^, ^27^ and is associated with greater WMH volumes.^28^ A recent review of antihypertensive therapy and cerebral small vessel disease trials showed that antihypertensive therapy protects against progression of WMHs.^29^ That we show attenuation of the risk of both VD and WMH volume on adjustment for greater use of antihypertensive medication in hyperglycaemic states can superficially be interpreted as treatment having adverse, not beneficial, effects. However, we suggest that in this context, receipt of antihypertensive medication acts as an indicator of longstanding untreated elevated blood pressure and that, therefore, treatment is being instituted too late. This is supported by a recent study that suggests that treatment for hypertension should begin as early as the third decade to potentially reduce risk of disease and early mortality.^30^ Early adulthood blood pressure, measured at around the age of 43 years, is also more strongly related to WMH volumes at age 70 than blood pressure measured throughout middle age, or indeed contemporaneous with WMH volume assessment.^31^ This serves to highlight the importance of elevated blood pressure, even before middle age. The role of even modest elevations in blood pressure, blood pressure trajectories from young adulthood, and early blood pressure‐lowering intervention, requires exploration in the context of reducing the risks of brain pathology.

We show associations between hyperglycaemic states, from prediabetes to established diabetes and all of our outcomes, with the exception of all‐cause dementia, for which excess risks only emerged in relation to known diabetes. Individuals with diagnosed, and thus treated, diabetes had lower HbA1c levels than the undiagnosed group, which is expected. Those with established diabetes have elevated HbA1c levels for around 10 years before diagnosis. Long‐term elevation of HbA1c levels is probably associated with poorer brain health. Hyperglycaemic states appeared to be associated somewhat more strongly with VD and WMH volume than AD and hippocampal volume, as the latter were resistant to adjustment for CVD risk factors. This is in line with evidence that diabetes is associated with greater WMH volume^11^, ^12^; also, the Genetics of Diabetes Audit and Research in Tayside Scotland (GoDARTS) case‐control study showed that those individuals with diabetes had a greater than twofold excess risk of VD, but no association with AD.^32^ Discrimination between VD and AD remains challenging and, to date, no studies have investigated the associations between lesser hyperglycaemic states and VD/AD in a single study.

That we observed a stronger association between glycaemia and VD and WMHs, as opposed to hippocampal volume and AD, is perhaps suggestive of two distinct, yet related neurological and vascular pathways. This is, in turn, supportive of a ‘two‐hit hypothesis’, which has recently grown in popularity.^33^ Briefly, a combination of genetic, environmental and vascular risk factors results in neurovascular dysfunction, alongside damage to arterioles, small arteries and brain capillaries, either through pathways independent of amyloid‐ß (hit one) and/or pathways dependent on amyloid‐ß (hit two). These pathways converge on blood vessels and can synchronously, or independently, cause the neuronal dysfunction associated with dementia.^33^ Just how these pathways act synergistically or independently remains unclear.

In the 18,809 participants who had follow‐up visual memory data we found that prediabetes and known diabetes conferred 42% and 39% excess risks of cognitive decline on multivariate adjustment, respectively. While only 32 people with prediabetes and 42 people with known diabetes experienced cognitive decline during the study follow‐up, the fact that both hyperglycaemic states were associated with adverse effects on brain health compared with normoglycaemic individuals adds confidence to our conclusion that hyperglycaemia negatively affects cognitive function, in line with previous observations.^34^ We observed that adjustment for BMI substantially increased the ORs from our demographics‐only model, such that individuals with higher BMI values were less probable to experience cognitive decline. This may relate to the ‘obesity paradox’, whereby those with higher BMI values have lower mortality rates than normal‐weight individuals, for which several explanations have been proposed.^34^ Importantly, once diagnosed, diabetes remains a lifelong condition and these individuals are at an increased risk of complications. However, while higher BMI in midlife is associated with a greater risk of cognitive decline, the reverse occurs in older age, supported by evidence of an inverse relationship between BMI and dementia mortality.^35^ The explanation is that weight loss occurs as a result of chronically ill health.^36^

Our study possesses some important strengths. The UKB is one of the largest studies to have data on HbA1c across the entire glycaemic spectrum, cognitive function, dementia subtypes and neuroimaging measures. We used validated algorithms to define diabetes and dementia, but we acknowledge that a completely accurate diagnosis of dementia, in particular, remains a challenge. The algorithm used to define dementia in the UKB was most accurate for all‐cause dementia, followed by AD then VD.^18^ The visual memory test used for follow‐up (and thus, to define cognitive decline) did not show good reliability (r = 0.16) in the UKB. The UKB had a low response rate and, consequently, may suffer from selection bias,^37^ which could mean that participants were less probable to have cognitive problems at study inception. Thus, it is possible that the association between glycaemia and our outcomes may have been underestimated.

In conclusion, we show that both prediabetes and known diabetes are detrimental in terms of VD and cognitive decline risk, which appear to be driven by treated hypertension. Somewhat weaker associations with all‐cause dementia and AD indicate that pathological mechanisms beyond standard CVD risk factors may affect brain health, in association with hyperglycaemia. Our findings of low‐normal HbA1c levels being associated with favourable WMH and hippocampal volumes are intriguing and require further investigation.

## CONFLICT OF INTEREST

KB reports grants from Diabetes UK, and grants from the British Heart Foundation (BHF), during the conduct of the study; and grants from the Medical Research Council (MRC), outside the submitted work. LS reports grants from the BHF and Diabetes UK during the conduct of the study; grants from Wellcome, the MRC, NIHR, GSK and the BHF, outside the submitted work; and is a Trustee of the BHF. NC reports grants from Diabetes UK, and grants from BHF, during the conduct of the study; and personal fees from AstraZeneca, and grants from the MRC, outside the submitted work. The remaining authors declare that they have no conflicts of interest to declare.

## AUTHOR CONTRIBUTIONS

Literature search: VG; study design: VG and NC; data analysis: VG and SVE; data interpretation: VG, NC, LS and KB; writing the manuscript: VG and NC; commenting on the draft manuscript: VG, A‐EF, SVE, RM, CTR, KB, LS and NC. VG guarantees the work carried out, had access to all of the data and takes responsibility for the integrity of the data and the accuracy of the data analysis.

### PEER REVIEW

The peer review history for this article is available at https://publons.com/publon/10.1111/dom.14321.

## Supporting information


**Appendix S1**: Supporting information

## Data Availability

The UK Biobank data are publicly available to all bona fide researchers at https://www.ukbiobank.ac.uk.
