# Supplementary material for: HbA1c and brain health across the entire glycaemic spectrum
Source: Diabetes Obes Metab. 2021 Feb 14;23(5):1140–9. doi: 10.1111/dom.14321 (PMC8261644; doi:10.1111/dom.14321)
Supplement: Supplementary file 1 — Appendix S1: Supporting information [file DOM-23-1140-s001.docx]

**Supplementary material**

*Details of the UK Biobank recruitment process*

Nine million men and women from across the UK were invited to take part in the study and thus, the response rate was 5.5%^1^. However, UKB set out to recruit 500,000 individuals, as detailed in the protocol^2^. Participants were assessed at baseline throughout 22 centres across the country and the visit included a self-completion touch-screen questionnaire, a computer-assisted interview, physical and functional phenotypes, and participants had blood, saliva and urine^3^. Participants have detailed phenotype data on physical (subjective and objective), mental and lifestyle measures, as well as linkages to routinely collected data (e.g. deaths, hospital admissions and cancer registers).

*Details of the UK Biobank HbA_1c_ protocol*

HbA_1c_ assays were performed using five Bio-Rad Variant II Turbo analysers, manufactured by Bio-Rad Laboratories, Inc. and employ a High Performance Liquid Chromatography (HPLC) method. A validation study ensured that the analysers underwent a multi-instrument comparison to ensure that they were in agreement^4^. More details are outlined in detail in the UK Biobank HbA_1c_ protocol^4^.

*Details of the UK Biobank neuroimaging protocol*

Briefly, participants were scanned across four centres in Central, North, South-East and South-West England. A centralised team was in charge of training and monitoring quality assurance across all four centres, with all staff members having undergone extensive training with an MR physicist. Harmonisation of data across centres is assured by employing the same scanner models, software, adjustment and tuning techniques, coil types and protocols. In addition, a standardised training programme is provided for radiographers in each centre and standard operating procedures, alongside phantom measurements, servicing and performance checks that are conducted by a UKB physicist. Qualitative and quantitative comparisons are performed by external imaging experts to confirm that images are of high quality and suitable for research.

*All-cause dementia in UKB*

The sum of AD and VD cases in our study is not equal to the number of all-cause dementia cases. This is because we included frontotemporal dementia in the all-cause dementia analyses and all-cause dementia also includes any other type of dementia (including unspecified dementia and dementia in other diseases classified elsewhere), as set out in the algorithmic paper by Wilkinson and colleagues^5^.

*Pre- and known diabetes and incident dementia – additional analyses to understand confounding by age*

To confirm that the associations between pre- and known diabetes and incident dementia were not due to these groups’ higher average age in comparison to the normoglycaemic group (Table 1), we performed Cox regressions with incremental adjustments to show confounding by age more clearly (Supplementary Table S1). These models indicate that while age is an important factor in these associations, our results remained strong and robust after its inclusion.

Table S1. *Crude, age + sex-adjusted and multiply-adjusted associations between pre-diabetes/known diabetes and incident dementia*

| Outcome | Crude | Age + sex-adjusted | Multiply-adjusted |
| --- | --- | --- | --- |
| *Prediabetes* | HR (95%CI) | HR (95%CI) | HR (95%CI) |
| All-cause dementia | 1.55 (1.27;1.89) | 1.26 (1.03;1.53) | 1.15 (0.94;1.41) |
| Vascular dementia | 2.38 (1.61;3.51) | 1.84 (1.25;2.73) | 1.54 (1.04;2.28) |
| *Known diabetes* | HR (95%CI) | HR (95%CI) | HR (95%CI) |
| All-cause dementia | 2.22 (1.95;2.54) | 1.91 (1.66;2.21) | 1.91 (1.66;2.21) |
| Vascular dementia | 5.56 (4.34;7.11) | 4.01 (3.13;5.15) | 2.97 (2.26;3.90) |
| Alzheimer’s dementia | 2.47 (1.97;3.10) | 1.96 (1.56;2.46) | 1.84 (1.44;2.36) |

*Note.* Crude=unadjusted, multiply-adjusted=adjusted for age + sex + deprivation + ethnicity + educational attainment + BMI + CVD + statins + antihypertensives + smoking; results for pre-diabetes and Alzheimer’s dementia are not presented, as we found no evidence of an association.

REFERENCES

1. Swanson JM. The UK Biobank and selection bias. Lancet 2012;380(9837):110.

2. UK Biobank. UK Biobank: Protocol for a large-scale prospective epidemiological resource. UKBB-PROT-09-06 (Main Phase) 2007;06(March):1–112.Available from: https://www.ukbiobank.ac.uk/wp-content/uploads/2011/11/UK-Biobank-Protocol.pdf

3. Sudlow C, Gallacher J, Allen N, et al. UK Biobank: An Open Access Resource for Identifying the Causes of a Wide Range of Complex Diseases of Middle and Old Age. PLoS Med. 2015;12(3):1–10.

4. Tierney A, Fry D, Almond R, et al. UK Biobank Biomarker Enhancement Project Companion Document to Accompany HbA1c Biomarker Data. 2018;1–8.Available from: https://biobank.ndph.ox.ac.uk/showcase/showcase/docs/serum_hb1ac.pdf

5. Wilkinson T, Schnier C, Bush K, et al. Identifying dementia outcomes in UK Biobank : a validation study of primary care , hospital admissions and mortality data. Eur. J. Epidemiol. 2019;34(6):557–565.
